# Pan-cancer functional analysis of somatic mutations in G protein-coupled receptors

**DOI:** 10.1038/s41598-022-25323-x

**Published:** 2022-12-13

**Authors:** B. J. Bongers, M. Gorostiola González, X. Wang, H. W. T. van Vlijmen, W. Jespers, H. Gutiérrez-de-Terán, K. Ye, A. P. IJzerman, L. H. Heitman, G. J. P. van Westen

**Affiliations:** 1 Division of Drug Discovery and Safety, Leiden Academic Centre for Drug Research, Leiden University, Leiden, The Netherlands; 2 ONCODE Institute, Leiden, The Netherlands; 3 Janssen Pharmaceutica NV, Beerse, Belgium; 4 Department of Cell and Molecular Biology, Uppsala University, Uppsala, Sweden; 5 School of Electronic and Information Engineering, Xi’an Jiaotong University, Xi’an, China

**Keywords:** Cancer, Computational biology and bioinformatics, Drug discovery, Computational science, Cheminformatics

## Abstract

G Protein-coupled receptors (GPCRs) are the most frequently exploited drug target family, moreover they are often found mutated in cancer. Here we used a dataset of mutations found in patient samples derived from the Genomic Data Commons and compared it to the natural human variance as exemplified by data from the 1000 genomes project. We explored cancer-related mutation patterns in all GPCR classes combined and individually. While the location of the mutations across the protein domains did not differ significantly in the two datasets, a mutation enrichment in cancer patients was observed among class-specific conserved motifs in GPCRs such as the Class A “DRY” motif. A Two-Entropy Analysis confirmed the correlation between residue conservation and cancer-related mutation frequency. We subsequently created a ranking of high scoring GPCRs, using a multi-objective approach (Pareto Front Ranking). Our approach was confirmed by re-discovery of established cancer targets such as the LPA and mGlu receptor families, but also discovered novel GPCRs which had not been linked to cancer before such as the P2Y Receptor 10 (*P2RY10*). Overall, this study presents a list of GPCRs that are amenable to experimental follow up to elucidate their role in cancer.

## Introduction

Cancer is the second leading cause of death globally^1^. Research towards this multifactorial disease has expanded our knowledge significantly over the last two decades^2^, leading to public databases containing patient-derived data^3^. Cancer is typically the result of compounding mutations that transform healthy cells to malignant ones^4^. Previous work involving large scale mutational analysis picked up G Protein-coupled receptors (GPCRs) as the second most mutated class of proteins in the context of cancer after kinases^5^. Cancer cells are driven to proliferate and avoid the immune system. GPCRs have multiple functions in this process from increased growth (early stage) all the way to metastasis (late stage)^6^. Thus, any anomalies in GPCR functioning might be related to cancer growth. Another interesting property of GPCRs is that they are the most common drug target family with around 35% of drugs acting through a GPCR^7^, providing a diverse set of molecular tools to potentially combat cancer.

GPCRs consist of seven highly conserved transmembrane (TM) domains, typically harboring the ligand binding pocket for natural ligands, e.g. endogenous hormones or neurotransmitters. Human GPCRs are divided in several classes based on sequence similarity: A, B, C, D, F and T (as used on GPCRdb)^8,9^. The TM domains are connected via extra- and intracellular loops (ECL; ICL) displaying a lower degree of conservation. Most GPCRs also have an eighth TM domain that is connected by intracellular loop 4. The extracellular loops are known to also be involved in ligand recognition and activation, whereas the intracellular part of the receptor is linked to G protein recognition and activation. Finally GPCRs contain an N- and C-terminus which are also relatively little conserved between and within classes^9,10^.

In previous work, knock-down studies have been performed on several proteins to identify their role in the context of cancer, typically embarked upon after prior identification of the protein’s role in cancer^11,12^. One of the main reasons these in vivo studies are done is to identify whether a mutation is either a driver, providing a selective growth advantage and promoting cancer development, or a passenger mutation occurring coincidentally. Moreover, these studies provide insight whether a driver mutation is located on either an oncogene or a tumor suppressor gene^13^. The prioritization of point mutations for experimental characterization, when the role of the protein in cancer is still unknown, could accelerate the discovery of relevant oncogenic alterations.

Here, we focused on GPCRs in the context of cancer by using patient-derived data sets and specifically looked at trends and mutational patterns in this protein family. We performed a deeper investigation into several “motifs”, parts of the GPCR sequence that are conserved that contribute most to the stability and function of the GPCR^14–19^. Class-specific motifs and several broad differences between classes were also considered. Moreover, we provided a list of GPCRs with known small molecule ligands (including approved drugs), ranked by interest for follow-up using multi-objective ranking. They were ranked on mutational count, mutations in regions of interest, availability of in-house expertise, and ability to perform virtual screening (by QSAR). Finally, we exemplified our findings in a more in-depth analysis on C-C chemokine receptor type 5 (CCR5) to show the feasibility of our approach.

## Results

### Overview of datasets

Missense mutations in all GPCR human classes were collected from the GDC and 1000 Genomes datasets (Table 1). The GDC dataset contained more subjects than the 1000 Genomes set, but both were in the same order of magnitude based on missense mutation count. However, as fewer unique missense mutations were found in natural variance, most cancer-related mutations had a small frequency. To account for differences in the datasets’ number of data points, the mutation ratio per dataset was used instead of absolute mutation frequency in the subsequent comparative analyses (see “Methods”).

**Table 1:** Overview of the composition of the GDC and 1000 Genomes datasets.

	GDC dataset (v 22.0)			1000 Genomes dataset (2020)		
Total subjects	10,179			3202		
Total cancer types	53			n/a		
Missense mutations	2,129,235			2,943,276		

	Missense mutations in GPCRs					
Class	Total	Unique	Unique receptors	Total	Unique	Unique receptors
All class	45,902	40,431	394	43,884	24,237	396
Class A	26,342	23,122	284	20,528	11,454	286
Class B	10,745	9588	47	15,439	8814	47
Class B1	1499	1342	15	2174	1283	15
Class B2	9246	8246	32	13,265	7531	32
Class C	5592	4842	22	5273	2644	22
Class F	1155	1039	11	487	368	11
Class T	1675	1494	24	1639	719	24
Other GPCRs	393	346	6	518	238	6

### Two-entropy analysis

**Figure 1:**
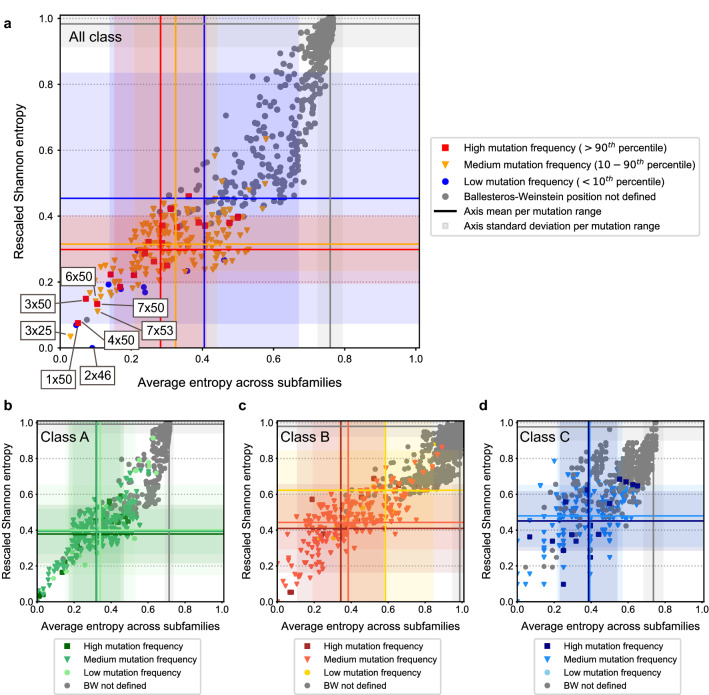
Shannon entropy across GPCR subfamilies versus Shannon global Entropy correlated to cancer-related mutations. A two-entropy analysis plot for all GPCRs with aligned positions. The average entropy across subfamilies (as defined by GPCRdb), i.e. conserved within a subfamily is on the x-axis, and the Shannon entropy on the y-axis. (**a**) Analysis for all GPCR classes combined. Residues are colored by the frequency of mutations found in the GDC dataset, with blue being low (< 10th percentile), orange medium (10–90th percentiles) and red high (> 90th percentile). Residues with no defined Ballesteros-Weinstein (BW) generic numbers are colored grey. Blue, orange, red, and grey lines represent the mean entropy values for each axis per mutation range (high, medium, low, and non-defined Ballesteros-Weinstein, respectively). Blue, orange, red, and grey shadows represent the standard deviation to the mean entropy values for each axis per mutation range (high, medium, low, and non-defined Ballesteros-Weinstein, respectively). (**b**) Analysis for Class A GPCRs. (**c**) Analysis for Class B GPCRs. (**d**) Analysis for Class C GPCRs. The coloring scheme for panels (**b**)–(**d**) is equivalent to that of panel (**a**).

A two-entropy analysis (TEA) was performed on our dataset as was done previously^19^. This method was chosen primarily to evaluate residue conservation across GPCRs and within GPCR subfamilies. Secondarily, we tried to leverage its ability to define residue functional characterization. Of note, we performed this analysis not only for Class A GPCRs but for all classes defined in GPCRdb; together and independently. Key to the TEA approach is that for each alignment position the Shannon entropy, which measures the level of conservation of amino acid residues at a certain position in a multiple sequence alignment, is calculated both within a GPCR subfamily and within all GPCRs. Therefore, the combination of these can provide a measure for the position function. Multiple interesting groups were identified, such as residues relevant for receptor function/activation (type Q3). Type Q3 are positions with a low Shannon entropy both within GPCR subfamilies and for the entire GPCR superfamily, this high conservation is linked to involvement in GPCR-conserved working mechanisms. Separating the graph into quadrants (Q1–4), type Q3 residues are represented in the bottom left quadrant in Fig. 1. A second group are residues relevant for ligand recognition (type Q2), made up by residues that are conserved within subfamilies, but not within the GPCR superfamily. Hence, these are associated with ligand recognition that is specific and conserved within a given subfamily. Type Q2 residues, represented in the top left quadrant were less noticeable in the all-class TEA (Fig. 1a), since inclusion of a larger number of subfamilies led to an increase in the overall entropy. However, it was more obvious in Classes A–C (Fig. 1b–d). Finally, in the top right quadrant of the TEA plot a third group of residues, Q1, is represented that are conserved neither among all GPCRs nor GPCR subfamilies. These are more likely to have only a small implication in receptor functions.

Residue conservation was linked to absolute mutation count frequency per position with Ballesteros-Weinstein number in cancer patients (color coding in Fig. 1 and Supplementary Fig. S1). Residues with a high mutation frequency were defined as those above the 90th percentile in the distribution of mutation counts by position. Conversely, residues with a low mutation frequency were defined as those under the 10th percentile. Absolute mutation count was (anti)correlated with entropy (Fig. 1). We observed a trend where more conserved type Q3 residues (bottom left quadrant, low entropy) had a higher mutation rate in cancer compared to the less conserved Q1 residues (top right quadrant, high entropy). We illustrated this with the mean ± SD entropy overall and across families (Fig. 1 and Supplementary Table S1). In the all-class TEA (Fig. 1a), the low mutation range had mean entropy values of 0.45 ± 0.38 and 0.41 ± 0.27 (Shannon and Average entropy across families, respectively). The high mutation range had lower mean entropy values of 0.30 ± 0.10 and 0.28 ± 0.13, respectively. On the contrary, this trend was not observed on natural variance data from the 1000 Genomes dataset (Supplementary Fig. S2). There, mean entropy values for the low mutation range were 0.40 ± 0.30 and 0.33 ± 0.23, respectively; and 0.34 ± 0.08 and 0.39 ± 0.12, respectively, for the high mutation range. We observed an average downward shift in entropy values for highly mutated positions per subfamily (not in the overall Shannon entropy) and an upward shift for less frequently mutated positions. Combined this showed a pressure in the GDC data for mutations in subfamily-conserved positions at the expense of mutations in non-conserved positions. This trend was maintained across classes, although less marked for for Classes B and C, and supported by the fact that from the type Q3 residues highlighted in Fig. 1a, higher mutation frequencies were associated with the most conserved positions in TM domains 3, 4, and 7 (i.e. 3 × 50, 4 × 50, and 7 × 50). These are part of the “DRY” (TM3), “GWGxP” (TM4), and “NPxxY” (TM7) conserved GPCR functional motifs. The high amount of mutations in residues of these and other motifs was investigated further in section “Mutation patterns within functionally conserved motifs”. Overall, cancer mutation frequency was correlated with individual residue conservation, hence we investigated groups of residues as defined by GPCR domains to further explore cancer mutation patterns.

### Mutation rates over GPCR structural domains

**Figure 2:**
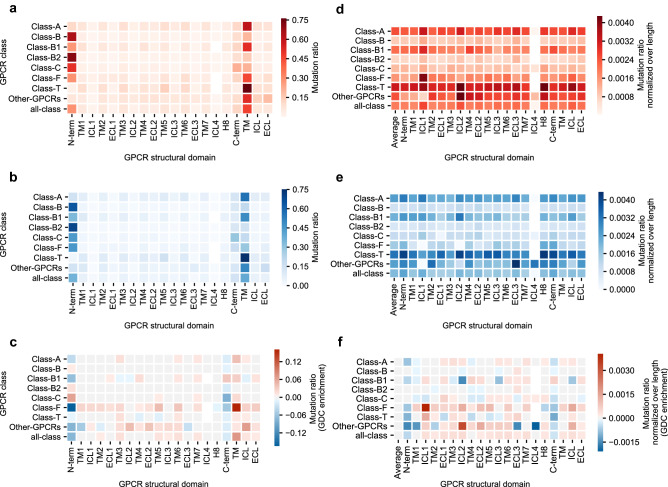
Distribution of mutation frequencies per GPCR structural domain. (**a**) Mutation ratio found in each structural domain in the GDC dataset for GPCRs in all classes combined and independently. (**b**) Mutation ratio found in each structural domain in the 1000 Genomes dataset for GPCRs in all classes combined and independently. (**c**) Mutation ratio enrichment in the GDC dataset over the 1000 Genomes dataset. (**d**) Mutation ratio normalized over average domain length found in each structural domain in the GDC dataset for GPCRs in all classes combined and independently. (**e**) Mutation ratio normalized over average domain length found in each structural domain in the 1000 Genomes dataset for GPCRs in all classes combined and independently. (**f**) Length-normalized mutation ratio enrichment in the GDC dataset over the 1000 Genomes dataset. “TM”, “ICL” and “ECL” represent the (normalized) mutation ratios in aggregated domains. In panels (**d**–**f**), “Average” represents the average ratio considering a domain as the whole protein. In panels (**a**) and (**d**), a darker shade of red represents a higher (normalized) mutation ratio in the GDC dataset. In panels (**b**) and (**e**), a darker shade of blue represents a higher (normalized) mutation ratio in the 1000 Genomes dataset. In panels (**c**) and (**f**), a darker shade of red represents a higher (normalized) mutation ratio enrichment towards the GDC dataset, while a darker shade of blue represents a higher (normalized) mutation ratio enrichment towards the 1000 Genomes dataset.

We hypothesized that mutations associated with altered function in the context of cancer, would occur more frequently in domains with higher conservation (i.e. TM domains) where positive selective pressure would favor them. Conversely, we expected mutations to be distributed more randomly over the sequence among the 1000 Genomes set and to be underrepresented in the conserved TM domains. However, the distribution in both sets was quite similar (Fig. 2a, b). Most mutations were in the N-terminus (~ 25% of the total across all classes), followed by the C-terminus (~ 15% of the total across all classes), which are on average the longest domains. The TM domains were next in mutation count, followed by ICL3 and ECL2. Finally, the remaining loops had the lowest amount of mutations. Around 40% of the mutations were found in the aggregated 7TM domains across all classes. No major differences between GDC and 1000 Genomes were observed when we compared mutation ratios (Fig. 2c), although there was enrichment observed in cancer-related mutations in the TM regions, as opposed to the N-terminus and C-terminus. To remove the bias caused by differences in average length of the different domains, we calculated the mutation ratio normalized over average domain length.

After normalization mutation ratios were more consistent over domains for every class in both the GDC and 1000 Genomes datasets (Fig. 2d, e). This correction was crucial to compare classes as observed in the N-terminus: Class B2 had a higher mutation ratio than Class T (Fig. 2a) but after normalization (Fig. 2d) a hotspot appeared in Class T. In general, all domains were slightly enriched in the GDC data except N-terminus and C-terminus (Fig. 2f). Of note were the differences observed between classes. For example, ICL2 was enriched across all classes (except B1) and highly enriched in Class Other GPCRs. Conversely, Class B1 showed a cancer enrichment in C-terminus that was not observed in any other class. Zooming in to specific domains showed mutational hotspots in different classes that can result in a therapeutic advantage. We concluded that some domains may be more amenable to mutation in the context of cancer. To further investigate these incipient mutation patterns in protein domains, we proceeded to the analysis of previously identified motifs that have a conserved function in GPCRs and that were also highlighted in our two-entropy analysis.

### Mutation patterns within functionally conserved motifs

Several highly conserved motifs relevant for GPCR function are known in different classes. They are “DRY”, “CWxP”, and “NPxxY” in Class A; “GWGxP”, “RE”, and “PxxG” in Class B; “HETx” in Class B2; and the “R/K” mutational hotspot in Class F (Table 2). Point mutations in these motifs usually cause a disruption or change in function^14–18^. We therefore hypothesized that mutational pressure in these motifs would occur in cancer to disturb normal GPCR function. For direct comparison between motifs we calculated a mutation ratio normalized over motif length. As a reference, the average normalized mutation rates obtained over the whole GDC and 1000 Genomes datasets are shown.

**Table 2:** Investigated motifs, and their residues as noted by their generic residue numbering, both class-specific and Ballesteros-Weinstein.

Motif	Class	Generic residues (Class-specific)	Ballesteros-Weinstein generic residues
DRY	Class A	3.49, 3.50, 3.51^a^	3 × 49, 3 × 50, 3 × 51
CWxP	Class A	6.47, 6.48, 6.49, 6.50^a^	6 × 47, 6 × 48, 6 × 49, 6 × 50
nPxxY	Class A	7.49, 7.50, 7.51, 7.52, 7.53^a^	7 × 49, 7 × 50, 7 × 51, 7 × 52, 7 × 53
HETx	Class B	2.50, 3.50, 6.42, 7.57^b^	2 × 43, 3 × 46, 6 × 37, 7 × 53
RE	Class B	2.46, 8.49^b^	2 × 39, 8 × 49
GWGxP	Class B	4.49, 4.50, 4.51, 4.52, 4.53^b^	4 × 49, 4 × 50, 4 × 51, 4 × 52, 4 × 53
PxxG	Class B	6.47, 6.48, 6.49, 6.50^b^	6 × 42, 6 × 43, 6 × 44, 6 × 45
R/K	Class F	6.32^c^	6 × 36

^a^Class-specific generic residue numbering scheme: Ballesteros-Weinstein^8,54^.

^b^Class-specific generic residue numbering scheme: Wootten^8^.

^c^Class-specific generic residue numbering scheme: Wang^8^.

**Figure 3:**
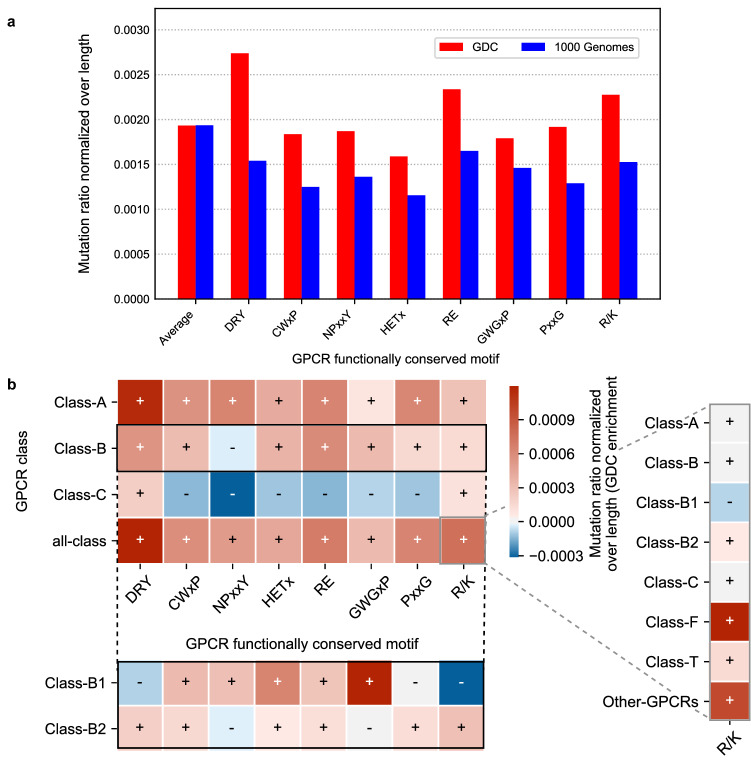
Distribution of mutation frequencies per functionally conserved motif. Mutation ratios normalized over motif length in GDC and 1000 Genomes datasets of conserved motifs found in different GPCR classes. Motifs analyzed are “DRY”, “CWxP”, and “NPxxY” (Class A); “HETx”, “RE”, “GWGxP”, and “PxxG” (Class B); and “R/K (Class F)”. “Average” represents the average ratio considering the whole protein length. (**a**) Analysis of all GPCR classes combined. Red bars show the normalized mutation ratio in the GDC dataset, while blue bars show the ratio of the 1000 Genomes dataset. (**b**) Length-normalized mutation ratio enrichment in the GDC dataset over the 1000 Genomes dataset in all classes combined and independently. The most populated classes are included in the main heatmap for visualization purposes. An extension of Class B is provided by breaking the heatmap row into Class B1 and Class B2. An extension of the all-class enrichment of the “R/K” motif is also provided for all classes independently. A darker shade of red represents a higher enrichment over the GDC dataset, and a darker shade of blue represents a higher enrichment over the 1000 Genomes dataset. Intensity of shades can be compared within the main heatmap (Classes A–C and all-class), and across each extension separately.

In each motif investigated the mutation rate in cancer patients was higher than the natural variation in that motif (Fig. 3a). Moreover, in the GDC dataset (red bars) “DRY”, “RE”, and “R/K” motifs were enriched in cancer compared to the average mutation ratio, whereas for the 1000 Genomes (blue bars) there was a clear reduction for all motifs. The GDC enrichment is shown for the most populated classes (Fig. 3b) and for all classes (Supplementary Fig. S3). Class A-specific domains (i.e. “DRY”, “CWxP”, and “NPxxY”) were enriched in Class A. Class B-specific domains (i.e. “HETx”, “RE”, “GWGxP”, and “PxxG”) were enriched mostly in Class B but also in Class A. Interestingly, the enrichment pattern was very different in Class B1 and B2. Of note, B2-specific motif “HETx” was more highly enriched for cancer mutations in Class B1. Finally, the “R/K” motif was slightly enriched in all classes except Class B1, but highly enriched in Class F. Class C showed minimal cancer enrichment across all motifs. An absolute count of the mutations found in the motifs in both sets is shown in Supplementary Fig. S4. We concluded that conserved motifs are increasingly mutated in cancer samples over natural variance, confirming their essential role and conservation.

**Figure 4:**
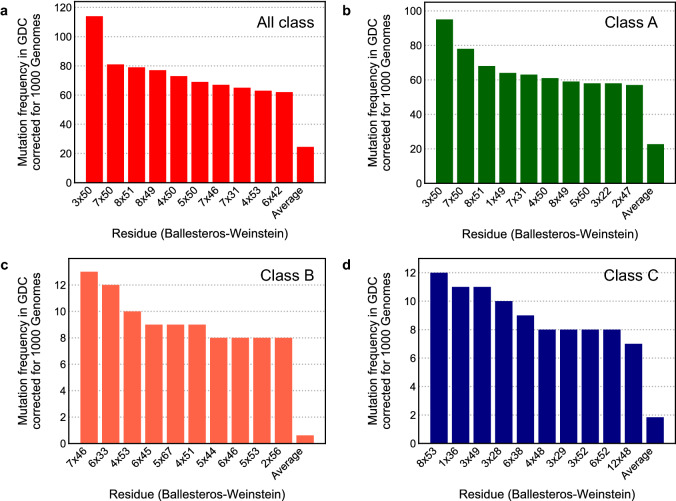
Most frequently mutated residues in GDC corrected for natural variance. The 10 positions with the highest mutation frequency in GPCRs in the GDC dataset corrected for the mutation frequency in the 1000 Genomes dataset. (**a**) Analysis of all GPCR classes combined. (**b**) Analysis of Class A GPCRs. (**c**) Analysis of Class B GCPRs. (**d**) Analysis of Class C GPCRs. The residue location in Ballesteros-Weinstein notation is shown on the x-axis, while on the y-axis the corrected mutation frequency of that residue is given. “Average” is the average mutation frequency per residue over all the data.

To gain further insights we selected the most mutated individual positions in the GDC dataset corrected for mutation frequency in natural variance. We represented this for all classes together and for Class A–C in Fig. 4. A count overview of unique GPCR cancer mutations is provided in Supplementary Fig. S5, and an overview of the substitutions found in all of the mutations in Supplementary Fig. S6.

Most of the mutations analyzed derived from Class A (Fig. 4), hence proving the relevance of a per class analysis. Overall and in Class A the most frequently mutated residue was 3 × 50 (BW numbering), part of the “DRY” motif. This was followed by 7 × 50 (“NPxxY” motif) in Class A. In Class B, 4 × 51 and 4 × 53 (“GWGxP” motif) and 6 × 45 (“PxxG” motif) were among the top 10. Interestingly, in Class A and Class C several residues in H8 were highly mutated (i.e. 8 × 49, 8 × 51, and 8 × 53), and in Class C we found an ICL1 residue (12 × 48) in the top 10. Given the enrichment in cancer found in functionally conserved motifs (Fig. 3), we suggest that the residues found among the most frequently mutated should be further functionally characterized since we hypothesize that they are relevant in receptor function.

### Ranking GPCRs for follow up

Having confirmed that patterns can be identified in GPCR mutations in cancer, we ranked GPCRs for experimental follow-up. Pareto sorting was performed to as a recommendation system to identify GPCRs with a suggested high impact in cancer biology amenable to small molecule intervention and follow up. Pareto sorting is based on multiple (not always correlating) properties. The Pareto analysis was done in two ways. Firstly, we implemented Pareto ranking solely based on somatic mutation data. The four selected properties for Pareto ranking were: Mutations in highly conserved TEA Q3 residues in GDC (maximized) and 1000 Genomes (minimized), and mutation rate in TM domains in GDC (maximized) and in 1000 Genomes (minimized). Additionally, we introduced two practical objectives to bias the mutation-based recommendation towards a set of in-house objectives representing feasibility of in vitro or in silico follow up. The feasibility of small molecule intervention was assessed by training a machine-learning model (random forest) for each GPCR in our data set using bioactivity data from ChEMBL 27, with circular fingerprints as molecular descriptors. The two practical objectives introduced were average R^2^ of ChEMBL QSAR prediction models (maximized), and in-house availability of proteins for experiments (maximized). The order of the properties determined the priority during the Pareto sorting.

**Figure 5:**
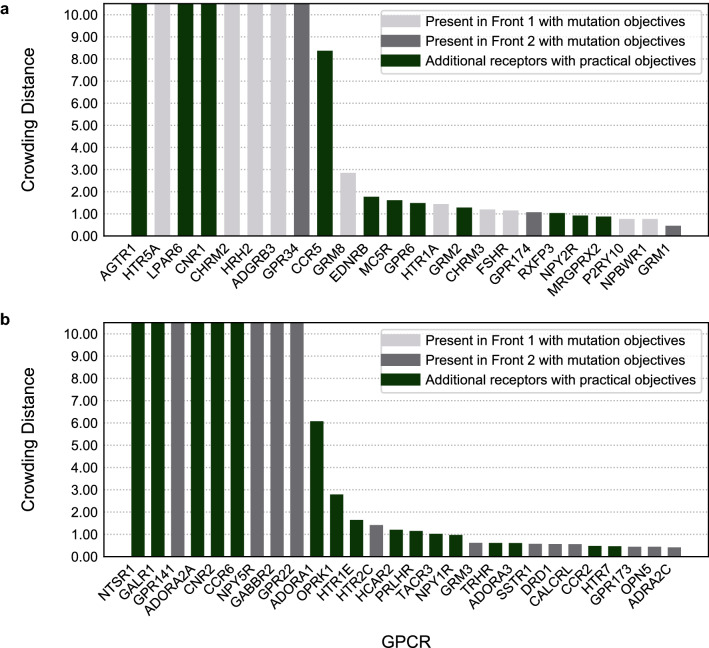
Crowding distances of the first and second Pareto fronts. (**a**) First Pareto front, consisting of 24 GPCRs. (**b**) Second Pareto front, consisting of 28 GPCRs. On the x-axis the gene names of GPCRs are shown, while on the y-axis their crowding distance is shown. Crowding distance was cut-off at 10, as the differences between these high-scoring receptors become negligible above that threshold. In grey, GPCRs detected by Pareto ranking using exclusively four mutation-derived objectives (light gray for the 1st front and darker grey for the 2nd front). In green, additional GPCRs that show up in the first two Pareto fronts by adding practical objectives to the recommendation system.

The first front In the Pareto optimization is considered “dominating”, which means that this set of GPCRs scored better in the combined properties than any other set. For the remaining data points a second front can be calculated, with GPCRs that scored worse than those in the first front but better than the rest of the solutions. Therefore, we used the first and second fronts for a subsequent ranking based on crowding distances between the receptors (Fig. 5a and b, respectively). Crowding distances are a measure of how dense the environment is; denser environments mean more balance in the objectives and thus more interesting GPCRs. As the crowding distance can go up to near infinite, we used a cut-off at a value of 10.

Twenty-four GPCRs from the best scoring (first) front translated to the GPCRs with the most desirable scores in the combined objectives of the Pareto optimization including “practical objectives” (Fig. 5a). The 13 receptors identified in the first front using exclusively mutation-derived objectives were contained in their totality in the first Pareto front with all objectives and, similarly, the 12 receptors in the mutation-only second front were entirely distributed between the first and second fronts (Fig. 5). GPCRs previously linked to cancer showed up in the first front alongside others that have not been thoroughly investigated yet. This was confirmed in a similar (non Pareto) ranking for GPCR subfamilies (Supplementary Fig. S7). The second Pareto front (Fig. 5b), contained 28 GPCRs. Hence, our recommendation system produced Pareto fronts that represented a list of potential candidates for follow-up experimental research. From the receptors of our first Pareto front we selected one for which there was in-house expertise, *CCR5*, as a case study for further investigation using a crystal structure based analysis to characterize the potential effects of the retrieved mutations in receptor function and/or ligand binding.

### CCR5 structural analysis

**Figure 6:**
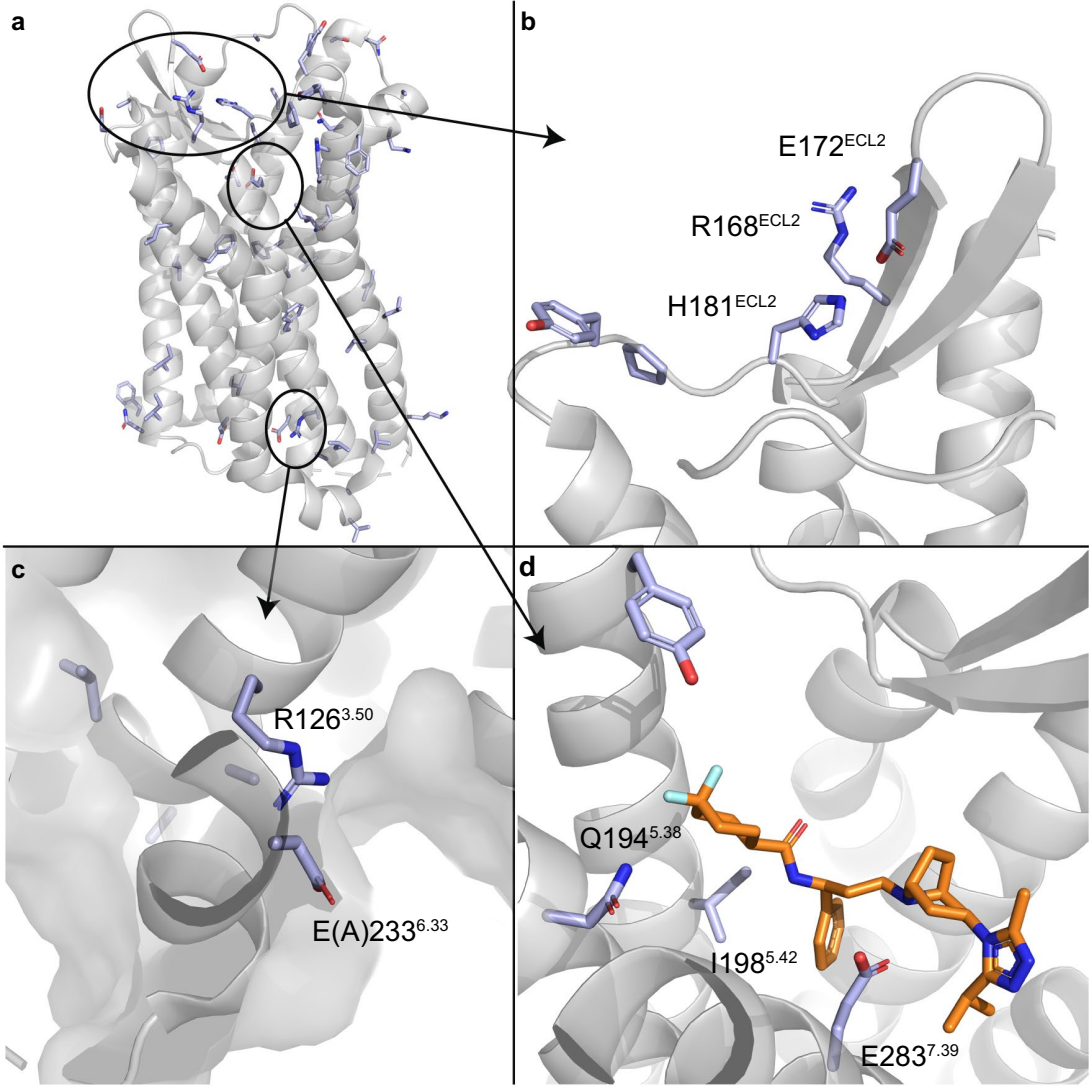
Cancer-derived mutation mapping in CCR5 structure. (**a**) The mutations found in the GDC dataset for CCR5 mapped on the 3D structure of the receptor. (**b**) Mutated residues found in ECL2 region. (**c**) G protein binding site, containing the mutation A233^6.33^E, which has been characterized as a thermostabilizing mutation for the inactive CCR5 structure (PDB code 4MBS). (**d**) The orthosteric binding site, with the small molecule inhibitor maraviroc (orange).

Mutations found in the GDC dataset for *CCR5* were cross-linked to GPCRdb data to find prior mutagenesis data. We then mapped the mutations onto the protein structure (PDB code 4MBS^20^). We focused on regions relevant for protein function and ligand binding. These mutations are widely spread across the receptor’s structure (Fig. 6a), including mutations in ECL2—a region that largely contributes to chemokine ligand recognition (Fig. 6b), G protein binding region (Figs. 6c), and orthosteric binding site (Fig. 6d). The crystal structure of *CCR5* used as a reference in Fig. 6 (PDB code 4MBS) contains the thermostabilizing mutation *A233*^*6.33*^*E*, which has been characterized for the inactive *CCR5* conformation*.* In this structure, a small molecule inhibitor—maraviroc—is co-crystalized in the orthosteric binding site (i.e. spanning the so-called major and minor binding pocket). Of note, some of the mutations found in the GDC dataset were in positions in close proximity to the inhibitor. Out of the 73 mutations found in our dataset, only 12 mutations had been previously annotated, while 37 mutations had no data available and 24 consisted of not-annotated data. Further analysis of previously annotated data shed some light on the functional implications of these mutations.

## Discussion

Here we performed a comprehensive comparison of mutations found in cancer patients (GDC dataset) versus mutations found in natural variance (1000 Genomes dataset) in all classes of GPCRs together and independently. We followed this up by investigating several highly conserved motifs for an increase in mutation rate compared to the other residues. Finally, we performed a Pareto Front analysis to create a ranking of GPCRs that warrant follow-up for their context in cancer, and we analyzed some of the cancer-related mutations found for one of the top-ranking receptors from a functional-structural point of view.

Our original hypothesis was that more conserved residues (i.e. lower entropy in a two-entropy analysis of all residue positions in the GPCRdb alignment) would experience a higher mutational pressure in cancer patients. We confirmed a trend for the all-class analysis showing that positions with a low amount of mutations per position were assigned higher entropy values than positions with a high amount of mutations per position (Fig. 1a). Conversely, the trend was not observed in a similar analysis in the 1000 Genomes dataset (Supplementary Fig. S2). Overall, we identified an incipient pattern between functional conservation and mutation rates in the GDC set, which was maintained in class-specific analyses thus confining the applicability domain of the TEA originally established by Ye et al.^19^. However, subfamily-specific residues were not identified in the all-class analysis, possibly due to discrepancies in subfamily classification in GPCRdb. Other methods could be used to better distinguish functional residues across GPCR classes that, for example, are not dependent on a fixed subfamily classification (e.g. TEA-O also defined by Ye et al.^19^) or define the classification levels on the fly (e.g. TreeDet^21^).

We then studied mutation distribution after aggregating residues by protein (Fig. 2) and subsequentely compared these across all available classes. The total count of mutations found in the larger and less conserved domains (i.e. C- and N-terminus) is higher as the chance of mutations occurring is therefore higher. However, when corrected for average length most of them showed similar mutation rates. Of note, mutations in TM, ICL, and ECL domains showed an enrichment in cancer patients, while the contrary was observed for the C- and N-terminus (Fig. 2f). The ICL and ECL domains are known to be important in receptor stabilization, signal transmission, and ligand and G protein recognition^22,23^. However, they also represent the most variable domains in terms of length and motif composition explaining the lack of consistent enrichment across GPCR classes in cancer in these domains. This also aligns with the observation that GPCR mutation rates were not homogeneously distributed among cancer types. For example some primary sites (e.g. Corpus uteri) showed a clear enrichment compared to others (see Supplementary Fig. S8). Literature confirms this distribution with the emphasis on specific residue changes that affect the entire function of the protein^24,25^.

A clearer pattern emerged In conserved motifs of GPCRs. We speculate that changes in these positions have a very high chance of disabling receptor function, supported by the observed higher mutation pressure in cancer compared to natural variance across classes (Fig. 3a). Thus, mutations might not be tolerated in healthy tissue but can be advantageous to cancer development. “DRY” mutations can decrease G protein coupling and recognition leading to reduced binding affinity of drugs^26^. For both mutations in “DRY” and “NpxxY” it has been shown that a decrease in ligand-receptor complex stability may occur, decreasing the response from the GPCR^27,28^. These motifs have been shown to be collectively involved in a conserved Class A GPCR activation pathway^14^. As expected, “HETx”, “RE”, “GWGxP” and “PxxG” all showed mutation enrichment in cancer in Class B GPCRs, but also in Class A GPCRs. These motifs are important for TM signaling, with those with a mutated motif showing loss of function^15^. The same principle is found for the mutational “R/K” hotspot, which is highly mutated in Class F GPCRs, serving as a switch for receptor activation^18^. Additionally, we found highly mutated H8 residues, in line with their recent identification as a functionally conserved motif in Class A GPCRs related to downstream signaling^29^.

Subsequently, we ranked individual GPCRs for follow up work via Pareto front analysis (Fig. 5). Several of the top ranked receptors had a known link to cancer. Notable entries include the C-C Chemokine receptor (CCR) type 5, which has been linked to regulatory T cells mediating tumor growth^30^, and CCR type 2, a key player in microenvironment-derived tumor progression^31^, LPA (Lysophosphatidic acid) receptor *LPAR6*, upregulated in bladder cancer^32^, GRM (Metabotropic glutamate) receptors 2 (*GRM2*) and 8 (*GRM8*), known for dysregulating signaling pathways that are crucial in cancer prevention^33^; serotonin receptors 5HT_1A_ (*HTR1A*), known to be involved in at least breast, ovarian and pancreatic cancer, 5HT_5A_ (*HTR5A)*, recently linked to breast cancer^34,35^, and the adenosine A_1_ (*ADORA1*) and A_2A_ (*ADORA2A*) receptors, linked to the progression and metastasis of a variety of cancer types as well as immune escape and immunotherapy^36,37^. An example of a GPCR not previously linked directly to cancer was the P2Y receptor family member 10 (*P2RY10*), found in the first Pareto front. *P2RY10* has been linked to chemotaxis via eosinophil degranulation, which could make it a potential target in cancer, although this is still highly speculative^38^. Of note, cancer-related receptors were identified in our Pareto fronts both using exclusively somatic mutation-derived objectives and including practical objectives. The recommendation system proposed here is meant to allow user-specific objectives and therefore the practical objectives proposed here could be substituted by e.g. availability of crystal structures or cell-lines overexpressing the receptor of interest.

Finally, the structural analysis of site-mutagenesis data in one of the top receptors from the first Pareto front (*CCR5*) shed light into the functional implication of some of the cancer-related mutations. This included a cluster of six residues in ECL2 found within the GDC dataset, from which four positions were previously shown to influence chemokine binding when mutated to Ala^39,40^. In the G protein binding site, the Class A highly conserved R126^3^^.^^50^ was found to be mutated. This position is in the “DRY” motif and it is the most frequently mutated position in the GDC set, resulting in altered G protein coupling to the receptor in for instance the adenosine receptor family^41^. Some experimental evidence is available for *CCR5* as well, where mutation to Asn abolished G protein signaling^42^. In the orthosteric site, four amino acids were previously investigated by a site-directed mutagenesis study by Garcia-Perez et al., Y187^5.31^, I198^5.42^, N258^6.58^, and E283^7.39^^40^ with variable effects. Mutating residue E283^7.39^, to Ala or to the more conservative Gln, had the biggest effect on maraviroc affinity decrease. The structural effect of I198^5.42^ and E283^7.39^ mutations in maraviroc binding can be derived from the crystal structure of *CCR5* with this negative allosteric modulator (Fig. 6c). Mutations on these two positions had an important effect on the ligand binding of two other HIV-1 drugs—vicriviroc and aplaviroc—and clinical candidates—TAK-779 and TAK-220—in two studies^43,44^. Whilst E283^7.39^A abolishes maraviroc binding, chemokine CCL5 binding is mildly (20-fold) affected^43^. On the contrary, *Y187*^*5.31*^*A* showed almost no effect on the binding affinity of maraviroc, while affecting chemokine recognition^40^. These observations exemplify the relevance of our method to prioritize cancer-related mutations in site-mutagenesis studies, and link them to receptor activation, endogenous ligand recognition, and the recognition of small (drug-like) molecules.

While completing this manuscript the TCGA dataset was used to identify significantly mutated GPCRs in cancer in a complementary extensive study by Wu et al.^45^. In comparison, we elaborated on our findings through a motif analysis of highly conserved residues in GPCRs, a link to positional entropy, and a link to structural information (i.e. analyzing the *CCR5* chemokine receptor). Moreover, we included the availability of chemical tools to study the selected GPCRs, as exemplified by our QSAR models. Another recent study by Huh et al.^46^ focused on Class A GPCRs expressed in tumors reaching similar conclusions regarding Class A-specific functional motifs. There a similar method was used to calculate mutation enrichment from natural variance which predicted the impact of mutations in specific sequence positions. Their results were validated in vitro, confirming the parallel effect of Class A GPCR mutations in receptor signaling. Our results extend to all GPCR class-specific functional motifs, opening novel paths to GPCR cancer research. Recently, we have published analyses of two other GPCRs, the Adenosine A_1_ and A_2B_ receptors, for which cancer-related somatic mutations were identified similar to the analysis as presented here^47,48^. There we used a yeast system to explore the effect said cancer-related mutations have on receptor function directly and found that there is a complex pattern of activation modulation. Similar approaches could be used to experimentally validate the relevance in cancer of somatic mutations in across all GPCR classes prioritized in this work.

While here the focus was on GPCRs, other receptor families can be investigated in a similar manner. Notable examples include solute carriers, or receptor-tyrosine kinases. The objectives in the Pareto optimization can also be adapted, providing a modified way of scoring the receptors depending on the scope of the study. While our analysis focused on differences in missense mutations occurring in cancer patients and natural variance, many other alterations (e.g. insertion/deletions, gene and protein expression levels) have been reported for GPCRs in the context of cancer^6,49^, and complementary analyses could be executed focusing on these. Finally, this computational approach can become part of a targeted therapy pipeline, suggesting key locations for in vitro and in vivo cancer-associated studies.

## Conclusions

We conclude that mutations found in GPCRs related to cancer are in general weakly correlated to specific domains in the protein or functional conservation. However, there is a higher mutational pressure in class-specific functionally conserved motifs in cancer patients (as shown in the GDC set) compared to healthy individuals. Moreover, we show that the role and mechanism of specific mutations can be elucidated using structural analysis as an intermediate step towards experimental validation. Finally, we provide a list of GPCRs that are amenable to experimental follow-up. The data may help in exploring new avenues in the design of cancer therapies, either by linking existing data to ligand binding and recognition, or the identification of potential new roles for residues not previously studied.

## Methods

### Cancer related mutations

Cancer associated mutations were obtained from the Genomic Data Commons (GDC), part of the US National Cancer Institute effort (version 22.0, January 16th 2020)^3^. GDC contains multi-dimensional mapping of genomic changes in several cancer types, including the complete dataset from The Cancer Genomic Atlas project (TCGA)^50^. We re-compiled part of the GDC database version 22.0 in a MySQL format to facilitate reproducible, version-consistent, big data cancer data analysis. Data was obtained from the GDC API engine and data transfer tool, depending on availability (unrestricted-access data only). The SQL database contains 19 tables distributed in eight different fields. Some data fields (i.e. gene expression data) contain analyzed data derived from GDC raw data files. A more extensive description of the database architecture, analyses performed, and the end-to-end mapping strategy is available in the supplementary data. We used data on somatic missense mutations found in a diverse set of cancer types, to which we will refer as the “GDC” data set.

### Natural variation

As reference we used the 1000 Genomes data^51^, including an additional data set released in 2020 by the New York Genome Center (NYGC). This is a dataset containing the natural variation of mutations in the genome. The dataset used in this study was obtained from Uniprot variance database in October 2020^52^. From this data, all somatic missense mutations were gathered. Subsequently, only mutations found in the 1000 Genomes subset were kept, removing cancer derived mutations from COSMIC and known pathological mutations. We refer to this dataset as “1000 Genomes”.

### Mutation dataset curation

We filtered both sets for GPCR-unique mutation pairs, along with the frequency. At the same time we annotated the resulting GDC and 1000 Genomes datasets with identifiers from GPCRdb^8^. This set was used for two entropy analysis, domain based analysis, and motif based analysis. Subsequently, prior to QSAR modelling and Pareto sorting, both datasets were enriched with bioactivity data from ChEMBL (release 27)^53^.

### Bioactivity data

From ChEMBL (release 27)^53^ ligand–protein interaction data was gathered for all GPCRs in GPCRdb^8^. Data points were filtered as follows: confidence score of 9, available pChEMBL value, and the protein belonging to the GPCR-family (L2 protein class). A pChEMBL value is a standardized value that equals to the negative logarithm of the measured activity for records with dose–response activity types.

### Structural information

The data set was enriched with structural information from GPCRdb^8^ for GPCRs present in the GDC and 1000 Genomes dataset. Included were the family trees to find related proteins, the amino acid sequence of a protein, and sequence alignment data to add generic numbering to the residues. Finally, we used the HUGO Gene Nomenclature Committee (HGNC) identifiers for source to source mapping.

### Multiple sequence alignment and generic numbering

The structurally supported multiple sequence alignment (MSA) provided by GPCRdb was used to study sequence conservation and link sequence positions to sequence- and structure-based generic GPCR numbering schemes. Generic numbering schemes (such as Ballesteros-Weinstein for Class A^54^) can be used to compare positions between GPCRs but are often limited to the TM domains. There are two parts to the numbereparatedd by a decimal sign. The first identifies the domain (e.g. TM), and the second is relative to the most conserved residue in that TM. The most conserved residue is defined to be position 50, with downstream positions receiving a lower number (towards the N-terminus) and upstream positions receiving a higher number (towards the C-terminus). Other schemes are available for Class B, C, and F. Structure-based curations of these schemes have been developed by GPCRdb^8^. The GPCRdb generic values contain the same two parts, but separated by an ‘x’ for differentiation purposes. We annotated the MSA with class-specific structure-based GPCRdb numbering schemes. Finally, we cross-linked the class-specific generic numbers with the more abundant class-A GPCRdb (GPCRdb(A)) equivalent to facilitate all-class analyses. For consistency, we refer to generic residue numbers in our work as Ballesteros-Weinstein, or BW, but give the GPCRdb(A) notation (i.e. 3 × 50 instead of 3.50) to denote the structural correction.

### Investigated motifs

Several conserved motifs commonly found in GPCRs were investigated (Table 2). All are found in literature to be functionally relevant in specific classes and often are referred to with the class-specific generic residue numbering schemes. To select these motifs across all classes, the Ballesteros-Weinstein residue numbering scheme was used.

### Two-entropy analysis

Two-entropy analysis (TEA) was performed as described previously in literature^19^. We reimplemented the revised TEA algorithm, adjusted by Ye et al., to account for gaps in the multiple sequence alignment and for the differences in number of subfamily members. The reimplementation was validated by application to the synthetic dataset provided in Ye et al. (Supplementary Fig. S9)^19^. We renamed “Total entropy” as “Rescaled Shannon entropy” and “Average entropy” as “Average entropy across subfamilies” for clarification. While the algorithm was not modified, two adaptations were made in the application, firstly using the GPCRdb hierarchy levels to define GPCR subfamilies, resulting in 83 subfamilies across all GPCR classes. From these, “Class A orphans” and “Class C orphans” were removed from the analysis. Secondly, we did not limit the entropy calculation to Class A GPCRS but applied it to all GPCR classes with more than one subfamily per class (Supplementary Table S2). However, contrary to previous work we included only human GPCR sequences.

### Statistical analysis per position

The frequencies of mutations in both sets were analyzed per class and in combination (Supplementary Table S2). Mutation frequency was calculated as the sum of patients bearing any unique mutation in any receptor in a position of the multiple sequence alignment included in:

- GPCR structural domains (i.e. N-terminus, TM domains, ECL and ICL loops, and C-terminus; also aggregated domains ‘TM’, ‘ECL’, and ‘ICL’)
- Functionally conserved motifs (Table 2)
- Individual alignment positions

To allow pairwise comparisons between sets, mutation ratios were calculated for cases (a) and (b), as defined in Eqs. (1)–(3):

1 $${\widetilde{M}}_{s,d}= \frac{{M}_{s,d}}{{M}_{s}}$$

2 $${\langle l\rangle }_{s,d}= \frac{\sum_{i=0}^{i={P}_{s,d}}{l}_{s,d,i}}{{P}_{s,d}}$$

3 $${\widetilde{M\mathrm{^{\prime}}}}_{s,d }= \frac{{\widetilde{M}}_{s,d}}{{\langle l\rangle }_{s,d}}$$

where ${M}_{s}$ is the mutation frequency in a set s, ${M}_{s,d}$ is the mutation frequency in a set s per domain d, ${\langle l\rangle }_{s,d}$ is the average length per set s and domain d, ${P}_{s,d}$ is the number of proteins per set s and domain d, and ${l}_{s,d,i}$ is the length (number of residues) per set s and domain d in a protein i.

The mutation ratio, ${\widetilde{M}}_{s,d}$, was visualized in Fig. 2a–c. The mutation ratio normalized over average domain length, ${\widetilde{M{^{\prime}}}}_{s,d}$, was visualized in Fig. 2d–f and in Fig. 3. In Fig. 2d–f, domains refer to GPCR structural domains and in Fig. 3 domains refer to functionally conserved GPCR motifs. In Figs. 2d–f and 3, a total mutation ratio, ${\widetilde{M}}_{s, d=total}$, was calculated for reference. This represents the average mutation ratio in one residue if the totality of the protein sequence is taken into account and in Figs. 2d–f and 3 is visualized as domain/motif “Average”. ${\widetilde{M}}_{s, d=total}$ and ${\widetilde{M{^{\prime}}}}_{s, d=total}$ are derived from Eqs. (1)–(3) as follows:

$${\widetilde{M}}_{s,d=total}= \frac{{M}_{s,d=total}}{{M}_{s}}=\frac{{M}_{s}}{{M}_{s}}=1$$

$${\langle l\rangle }_{s,d=total}= \frac{\sum_{i=0}^{i={P}_{s,d=total}}{l}_{s,d=total,i}}{{P}_{s,d=total}}$$

$${\widetilde{M\mathrm{^{\prime}}}}_{s,d=total }= \frac{{\widetilde{M}}_{s,d=total}}{{\langle l\rangle }_{s,d=total}}=\frac{1}{{\langle l\rangle }_{s,d=total}}$$

In Figs. 2c, d and 3b we calculated GDC enrichments by substracting ${\widetilde{M}}_{s=GDC,d}- {\widetilde{M}}_{s=1000 G,d}$ and ${\widetilde{M{^{\prime}}}}_{s=GDC,d }- {\widetilde{M{^{\prime}}}}_{s=1000 G,d}$, respectively.

For case (c) we calculated mutation frequency for each alignment position for the GDC and 1000 Genomes sets separately. Subsequently, we corrected the GDC frequency for natural variance by substracting the 1000 Genomes frequency from the GDC frequency.

### Pareto front

Multi-objective ranking was done within the Pareto method as implemented in Pipeline Pilot (version 18.1)^55^. Two implementations were designed. The first one was based exclusively on mutation data and the following properties were used: Mutation rate in TM domains in GDC (maximized), mutation rate in TM domains in the 1000 Genomes set (minimized), GDC mutations in highly conserved TEA Q3 residues (maximized), and 1000 Genomes mutations in TEA Q3 residues (minimized). For this purpose, TEA Q3 residues were defined as those in the all-class TEA with “Rescaled Shannon entropy” < 0.5 and “Average entropy across subfamilies” < 0.5. The second implementation included two practical objectives to bias the ranking towards recommendations for subsequent in vitro or in silico studies. These practical objectives were average R^2^ of ChEMBL QSAR prediction models (maximized), and in-house availability for experimental assays (maximized). The first and second front from each implementation were used in further analysis, but all data is provided as supporting information. The suitability of including practical objectives as part of a tunable recommendation system was evaluated by comparing the results of the two implementations. The performed QSAR models were Random Forest R models trained in Pipeline Pilot using 500 trees and a default seed of “12345”. A 50/50 percent training/ hold-out test set was used in duplicate to create and validate these models, with ECFP6 used as molecular descriptors^56^.

### 3D structural analysis

*CCR5* crystal structure (PDB code 4MBS) was obtained from the Protein Data Bank^20^. Mutagenesis data was retrieved from the GPCRdb and mapped onto the 3D crystal structure using PyMol^57^.

### Hardware

Sequence analysis, data processing, and QSAR modeling were run on a Linux server running CentOS 7. Hardware configuration: 2 × Intel Xeon Platinum 8160 (2.10), 48 cores, 256 DDR3 RAM, the jobs directory was located on a 1.6 TB PCIe SSD running in Raid-0.

### Software

Accelrys Pipeline Pilot 2018 (version 18) was used for all the calculations and analysis^55^. Any calculations performed were done in SI units, using the infrastructure provided in Pipeline Pilot. Data was written towards plain text files and Excel. Graphs were created using Python’s module Matplotlib^58^.

## Supplementary Information

Supplementary Information.

## Data Availability

The datasets and analysis code supporting the conclusions of this article are available in the 4TU repository https://doi.org/10.4121/15022410, including the mySQL GDC implementation. The source code used to produce the results in this manuscript was generated in the commercial software package Accelrys Pipeline Pilot 2018 (version 18). The 1000 Genomes dataset was obtained from UNIPROT ‘ftp://ftp.uniprot.org/pub/databases/uniprot/current_release/knowledgebase/variants/’. ChEMBL data was obtained from ‘https://www.ebi.ac.uk/chembl/’, GPCRdb data was obtained from ‘https://gpcrdb.org/’ and HGNC mapping data was obtained from ‘https://www.genenames.org/’.
