# Supplementary material for: Pan-cancer functional analysis of somatic mutations in G protein-coupled receptors
Source: Sci Rep. 2022 Dec 13;12:21534. doi: 10.1038/s41598-022-25323-x (PMC9747925; doi:10.1038/s41598-022-25323-x)
Supplement: Supplementary file 1 — Supplementary Information. [file 41598_2022_25323_MOESM1_ESM.pdf]

Pan-cancer functional analysis of somatic mutations in G protein-coupled receptors

SUPPLEMENTATY FIGURES AND TABLES

B.J. Bongers[1†], M. Gorostiola González[1,2†], X. Wang[1], H.W.T. van Vlijmen[1,3], W. Jespers[1,4], H. Gutiérrez-de-Terán[4], K. Ye[5], A.P. IJzerman[1], L.H. Heitman[1,2], G.J.P. van Westen[1*].

[1] Division of Drug Discovery and Safety, Leiden Academic Centre for Drug Research, Leiden University, Leiden, The Netherlands

[2] ONCODE Institute, Leiden, The Netherlands

[3] Janssen Pharmaceutica NV, Beerse, Belgium

[4] Department of Cell and Molecular Biology, Uppsala University, Uppsala, Sweden

[5] School of Electronic and Information Engineering, Xi'an Jiaotong University, Xi'an, China

* Corresponding author

Email: gerard@lacdr.leidenuniv.nl (GJPW)

[†] These authors contributed equally to this work

Supplementary table 1. ***Two-Entropy Analysis parameters for GDC and 1000 Genomes sets in all GPCR classes analyzed combined and independently. Shannon (Sh.) and Average group (Gr.) entropy mean and standard deviation (SD) values for all three levels of mutation rates: low (< 10th percentile), medium (10th -90th percentile), and high (>90th percentile).***

	**GDC**							**1000 Genomes**						
		**Low**<br>**Mean ± SD**		**Medium**<br>**Mean ± SD**		**High**<br>**Mean ± SD**			**Low**<br>**Mean ± SD**		**Medium**<br>**Mean ± SD**		**High**<br>**Mean ± SD**	
**Class**	**10th/90th percentiles**	**Sh.**	**Gr.**	**Sh.**	**Gr.**	**Sh.**	**Gr.**	**10th/90th percentiles**	**Sh.**	**Gr.**	**Sh.**	**Gr.**	**Sh.**	**Gr.**
**All class**	41/74	0.45 ± 0.38	0.41 ± 0.27	0.32 ± 0.08	0.32 ± 0.12	0.30 ± 0.10	0.28 ± 0.13	18/40	0.40 ± 0.30	0.33 ± 0.23	0.31 ± 0.09	0.31 ± 0.12	0.34 ± 0.08	0.39 ± 0.12
**Class A**	28/55	0.40 ± 0.25	0.34 ± 0.19	0.39 ± 0.13	0.32 ± 0.13	0.38 ± 0.16	0.32 ± 0.15	10/25	0.38 ± 0.22	0.28 ± 0.17	0.39 ± 0.14	0.32 ± 0.13	0.41 ± 0.10	0.38 ± 0.12
**Class B1**	1/5	-	-	0.41 ± 0.26	0.35 ± 0.30	0.39 ± 0.23	0.34 ± 0.28	1/5	-	-	0.42 ± 0.25	0.35 ± 0.29	0.53 ±0.26	0.49 ± 0.29
**Class B2**	3/9	0.53 ± 0.17	0.45 ± 0.21	0.46 ± 0.18	0.43 ± 0.21	0.43 ± 0.23	0.37 ± 0.22	2/9	0.43 ± 0.18	0.40 ± 0.20	0.47 ± 0.18	0.43 ± 0.21	0.41 ± 0.14	0.39 ± 0.12
**Class B**	4/13	0.62 ± 0.22	0.59 ± 0.26	0.44 ± 0.15	0.38 ± 0.19	0.41 ± 0.25	0.34 ± 0.24	3/13	0.52 ± 0.25	0.47 ± 0.26	0.45 ± 0.16	0.39 ± 0.2	0.46 ± 0.14	0.40 ± 0.14
**Class C**	1/6	-	-	0.48 ± 0.17	0.39 ± 0.18	0.45 ± 0.17	0.39 ± 0.16	1/4	-	-	0.50 ± 0.18	0.40 ± 0.19	0.50 ± 0.14	0.46 ± 0.11
**Class F ***	-	-	-	-	-	-	-	-	-	-	-	-	-	-
**Class T ***	-	-	-	-	-	-	-	-	-	-	-	-	-	-
**Other GPCRs ***	-	-	-	-	-	-	-	-	-	-	-	-	-	-

* Two Entropy Analysis was not performed in classes with only one GPCRdb subfamily defined.

Supplementary table 2. ***GPCR classes analyzed, number of members per class and GPCRdb subfamilies defined in the Two-Entropy Analysis.***

Class		Number of receptors in alignment	GPCRdb hierarchy levels (subfamilies)
All class		401	83
Class A (Rhodopsin)		289	61
Class B*		48	14
	Class B1 (Secretin)	15	5
	Class B2 (Adhesion)	33	9
Class C (Glutamate)		22	5
Class F (Frizzled)		11	1
Class T (Taste 2)		25	1
Other GPCRs		6	1

* Synthetic class formed by aggregation of Class B1 and Class B2 to facilitate the analysis of class-specific functional motifs described in the literature.

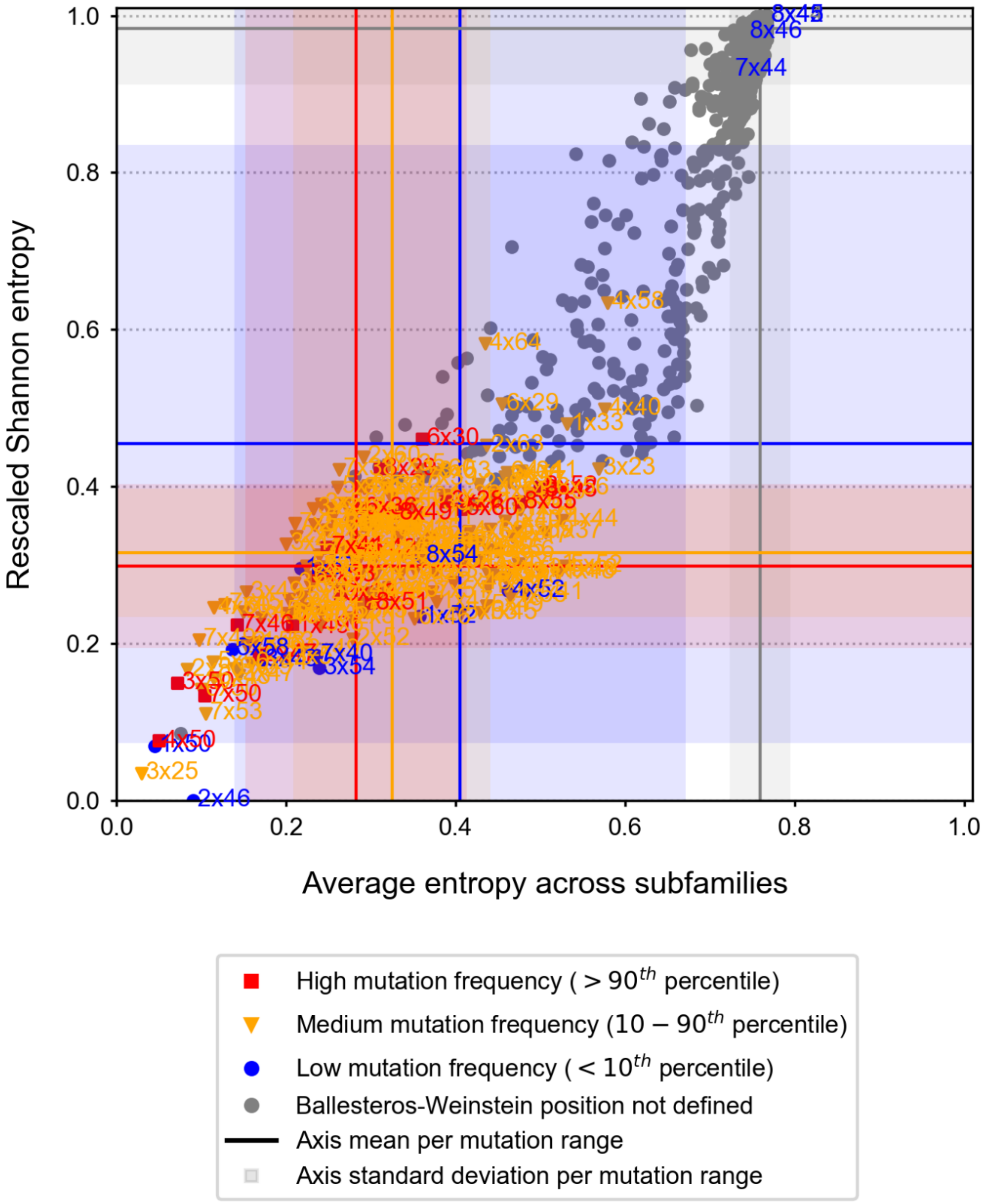

Supplementary figure 1. ***Shannon entropy across GPCR subfamilies versus Shannon global Entropy correlated to cancer-related mutations, with residue and GDC labels.*** *A two-entropy analysis plot for all GPCRs with aligned positions and labelled residues. The average entropy across families, i.e. conserved within a family is on the x-axis, and the Shannon entropy overall on the y-axis. Residues are colored by the frequency of mutations found in the GDC dataset, with blue being low (< 10th percentile), orange medium (10-90th percentiles) and red high (> 90th percentile). Residues with no defined Ballesteros-Weinstein labels are colored grey. Blue, orange, red, and grey lines represent the mean entropy values for each axis per mutation range (high, medium, low, and non-defined Ballesteros-Weinstein, respectively). Blue, orange, red, and grey shadows represent the standard deviation to the mean entropy values for each axis per mutation range (high, medium, low, and non-defined Ballesteros-Weinstein, respectively).*

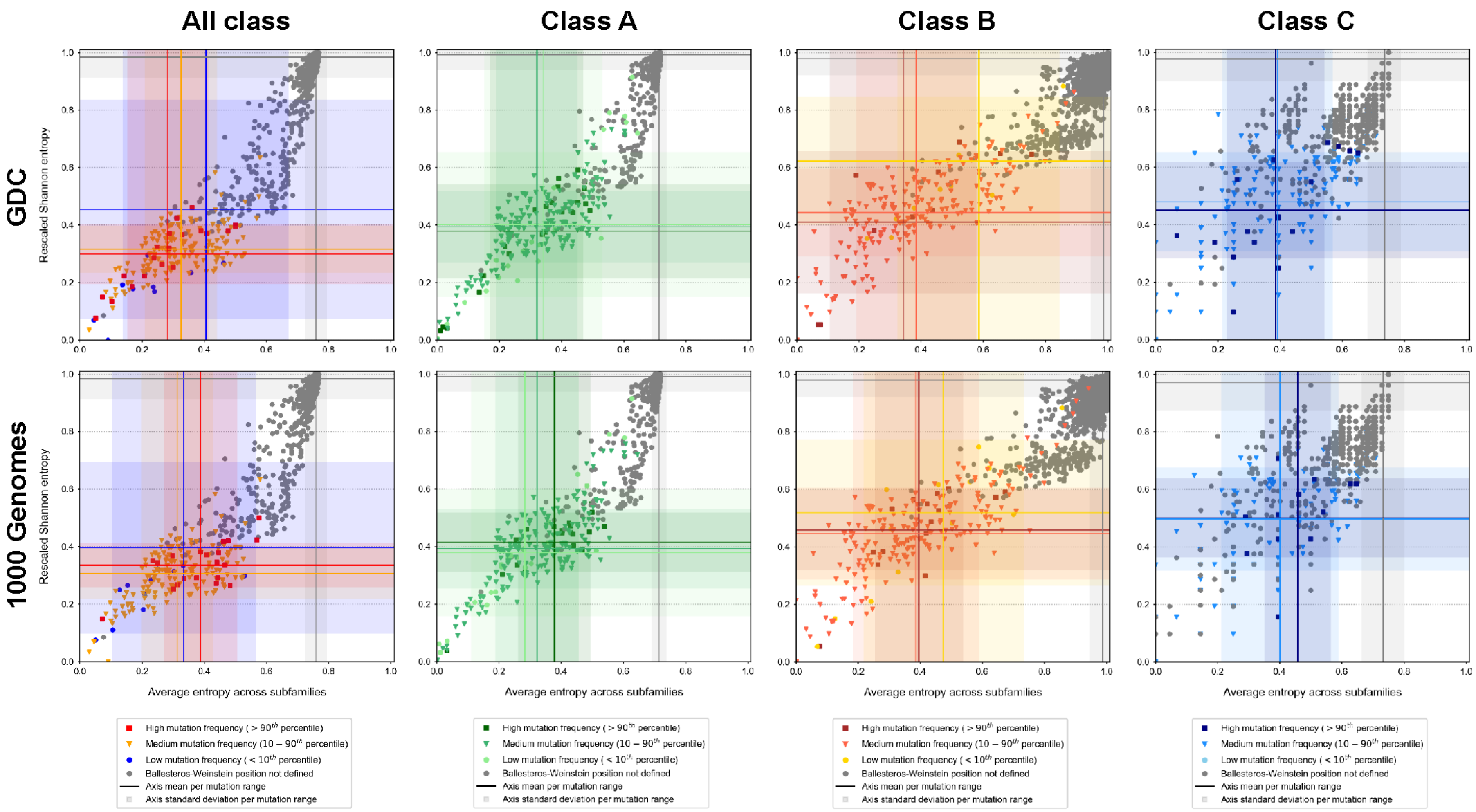

Supplementary figure 2. ***Two-entropy analysis correlated to cancer-related mutations and natural variance across GCPR classes.*** *The analysis is performed on all GPCR classes combined, as well as Class A-C independently. Residues are colored by the frequency of mutations found in the GDC dataset (top row), and the 1000 genomes dataset (bottom row). In the all-class analysis, blue is low (< 10th percentile), orange medium (10-90th percentiles) and red high (> 90th percentile) mutation frequency. Residues with no defined Ballesteros-Weinstein generic numbers are colored grey. Blue, orange, red, and grey lines represent the mean entropy values for each axis per mutation range (high, medium, low, and non-defined Ballesteros-Weinstein, respectively). Blue, orange, red, and grey shadows represent the standard deviation to the mean entropy values for each axis per mutation range (high, medium, low, and non-defined Ballesteros-Weinstein, respectively). The coloring scheme classes A-C is equivalent to that of all classes combined.*

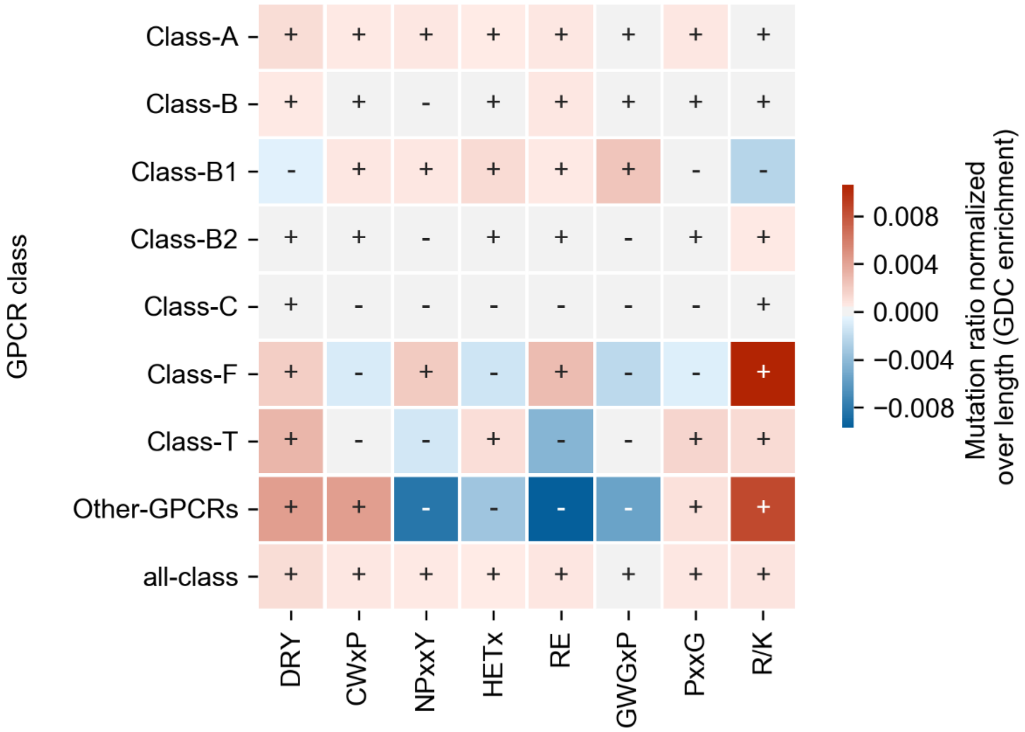

Supplementary figure 3. ***Enrichment of mutation frequencies per GPCR functionally conserved motifs across all GPCR classes.*** *Length-normalized mutation ratio enrichment in the GDC dataset over the 1000 Genomes dataset in all classes combined and independently. Motifs analyzed are "DRY", "CWxP", and "NPxxY" (Class A); "HETx", "RE", "GWGxP", and "PxxG" (Class B); and "R/K (Class F)". "Average" represents the average ratio considering the totality of the protein length. A darker shade of red represents a higher enrichment over the GDC dataset, and a darker shade of blue represents a higher enrichment over the 1000 Genomes dataset.*

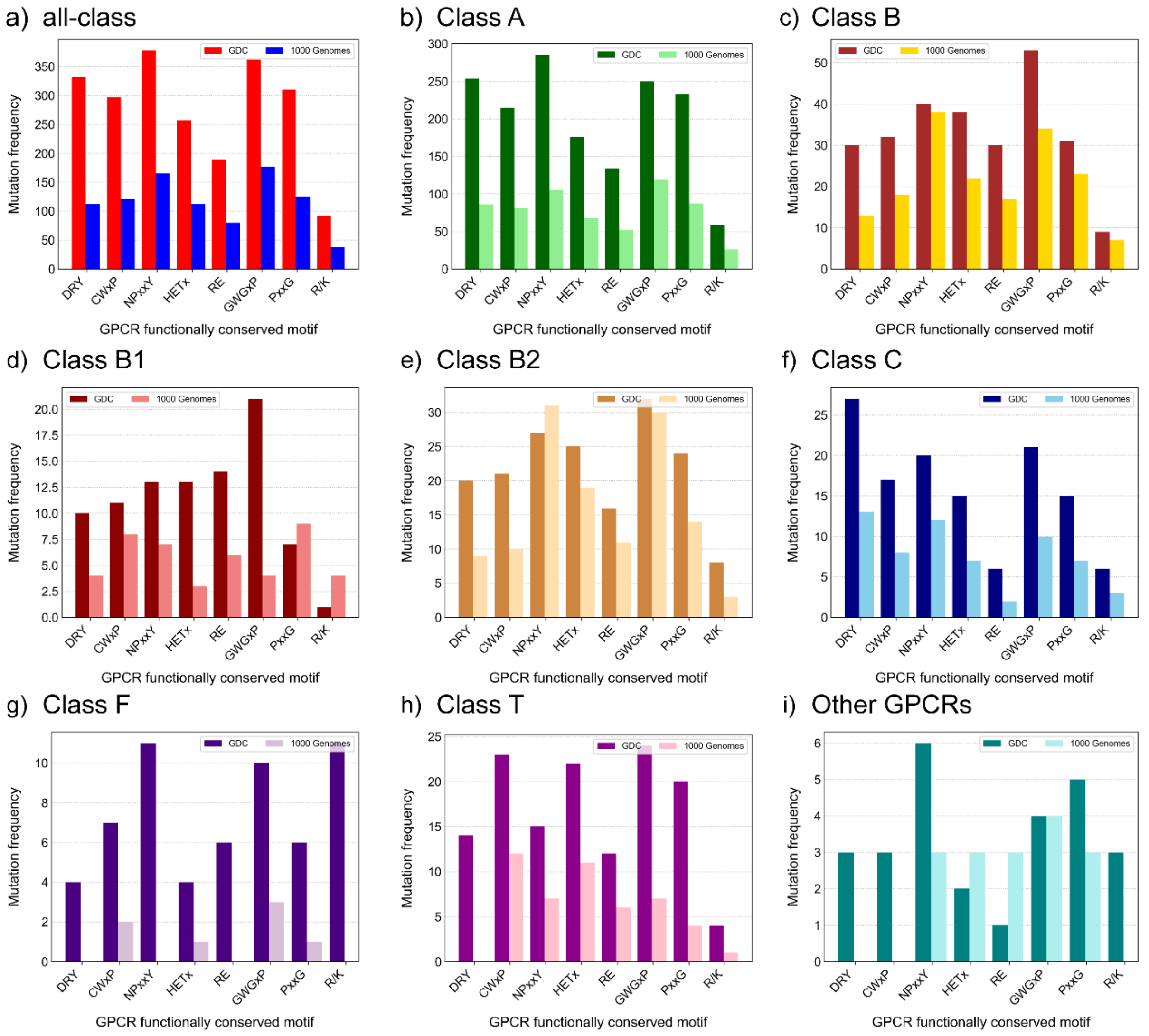

Supplementary figure 4. ***Mutation frequency cancer and natural variance in GPCR functionally conserved motifs across GPCR classes.*** *Motifs analyzed are "DRY", "CWxP", and "NPxxY" (Class A); "HETx", "RE", "GWGxP", and "PxxG" (Class B); and "R/K (Class F)".* ***(a)*** *Analysis of all GPCR classes combined.* ***(b)*** *Analysis of Class A.* ***(c)*** *Analysis of Class B.* ***(d)*** *Analysis of Class B1.* ***(e)*** *Analysis of Class B2.* ***(f)*** *Analysis of Class C.* ***(g)*** *Analysis of Class F.* ***(h)*** *Analysis of Class T.* ***(i)*** *Analysis of Class Other GPCRs.*

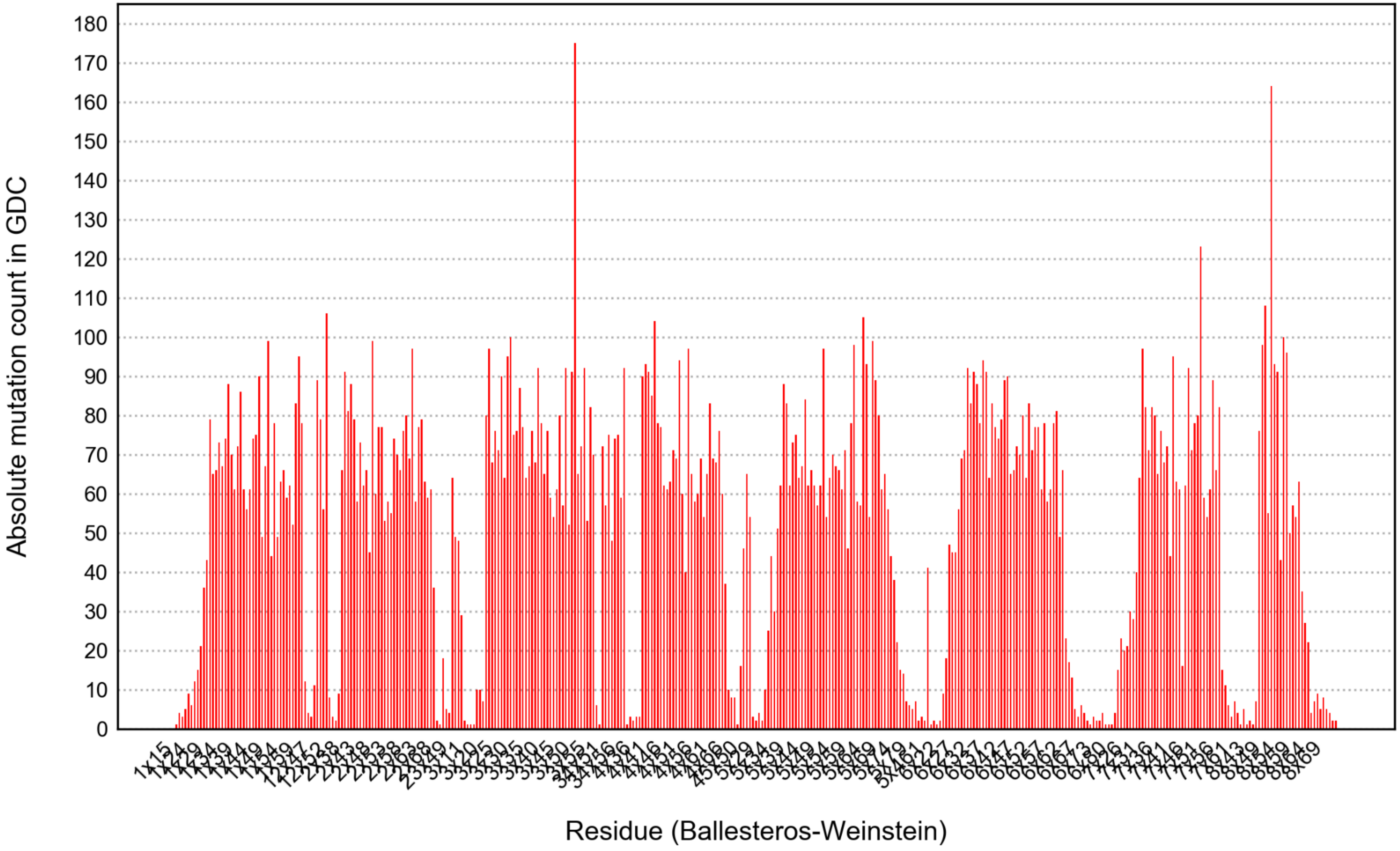

Supplementary figure 5. ***GPCR cancer mutations on Ballesteros-Weinstein positions.*** *GPCR cancer mutations plotted for the Ballesteros-Weinstein positions found in the GDC data. Positions are ordered from lowest to highest and X-axis labels are displayed every five residues for visualization purposes.*

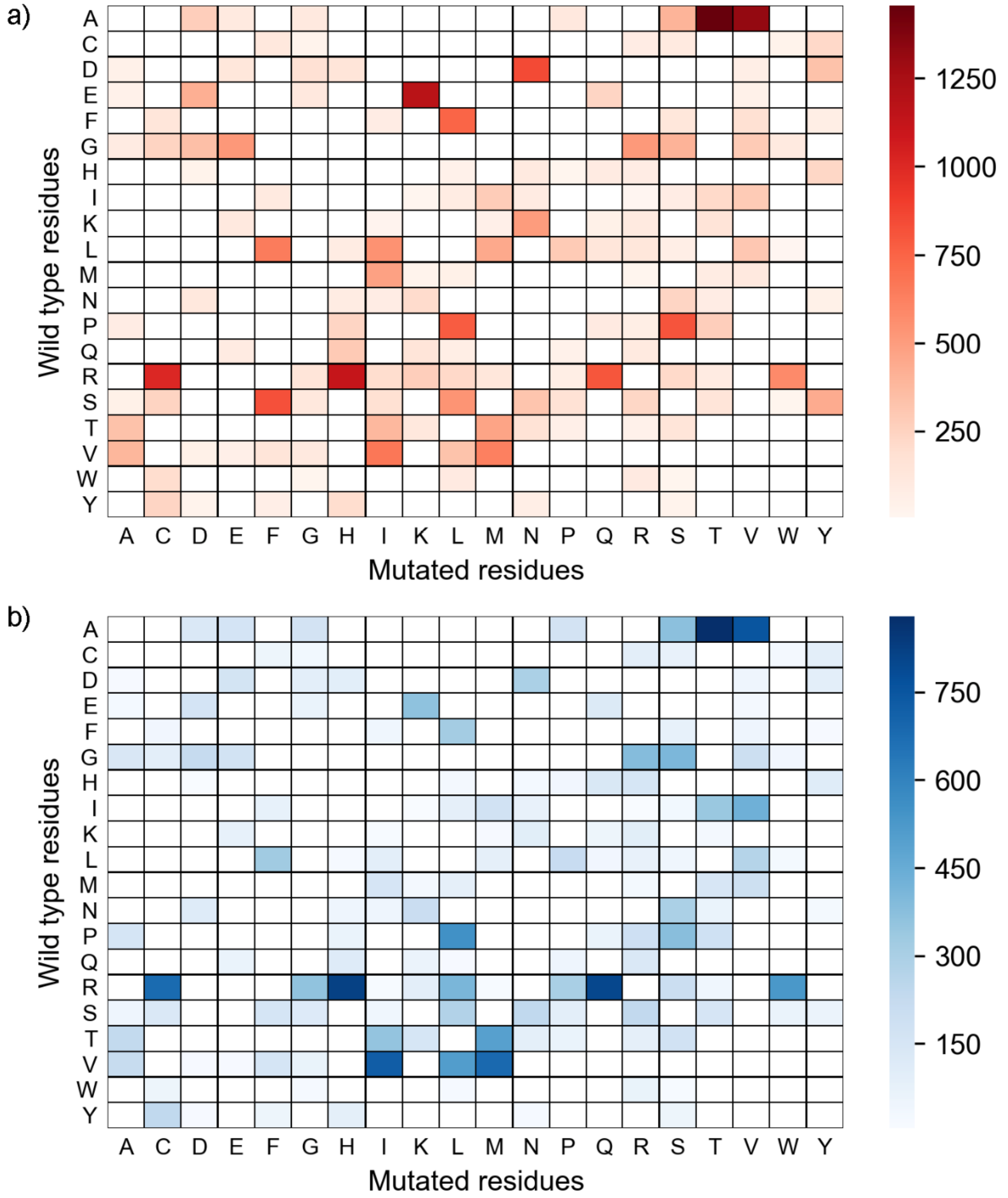

*Supplementary figure 6. **Heat-map cancer substitutions.** **(a)** Heat-map showing the frequency of substitutions found in the GDC dataset. A darker shade of red means a higher frequency. **(b)** Heat-map showing the frequency of substitutions found in the 1000 Genomes dataset. A darker shade of blue means a higher frequency*

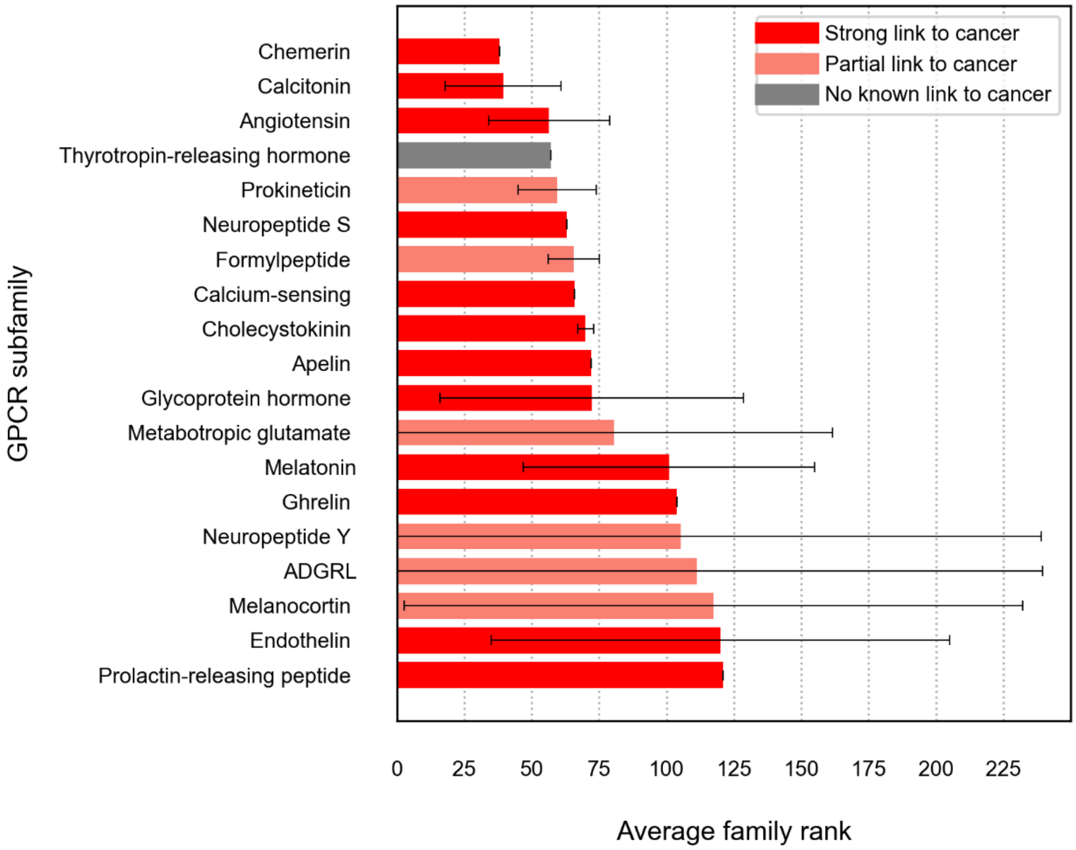

Supplementary figure 7. ***Average Rank of GPCR families and their link to cancer in the literature.*** *Average rank of GPCR families related to the mutation ratio in individual family members. For each GPCR, the absolute mutation count was divided by receptor length, to provide a mutation rate for each. To identify patterns within GPCR families, a family-wide rank was calculated by averaging the ranking of each of the members in a family and subsequently compared to the other families Shown on the y-axis are the different GPCR families as categorized by GPCRdb, while on the x-axis their average rank as a receptor family is given. The lower average rank value, the better. The error bars represent standard deviation of individual GPCR rankings within the family. Color-coding represent the link to cancer in the literature for the family. Red represents a strong link (i.e. all members of the family have been linked to cancer), salmon represents partial link (i.e. some members of the family have been linked to cancer), and grey represents no link to cancer reported.*

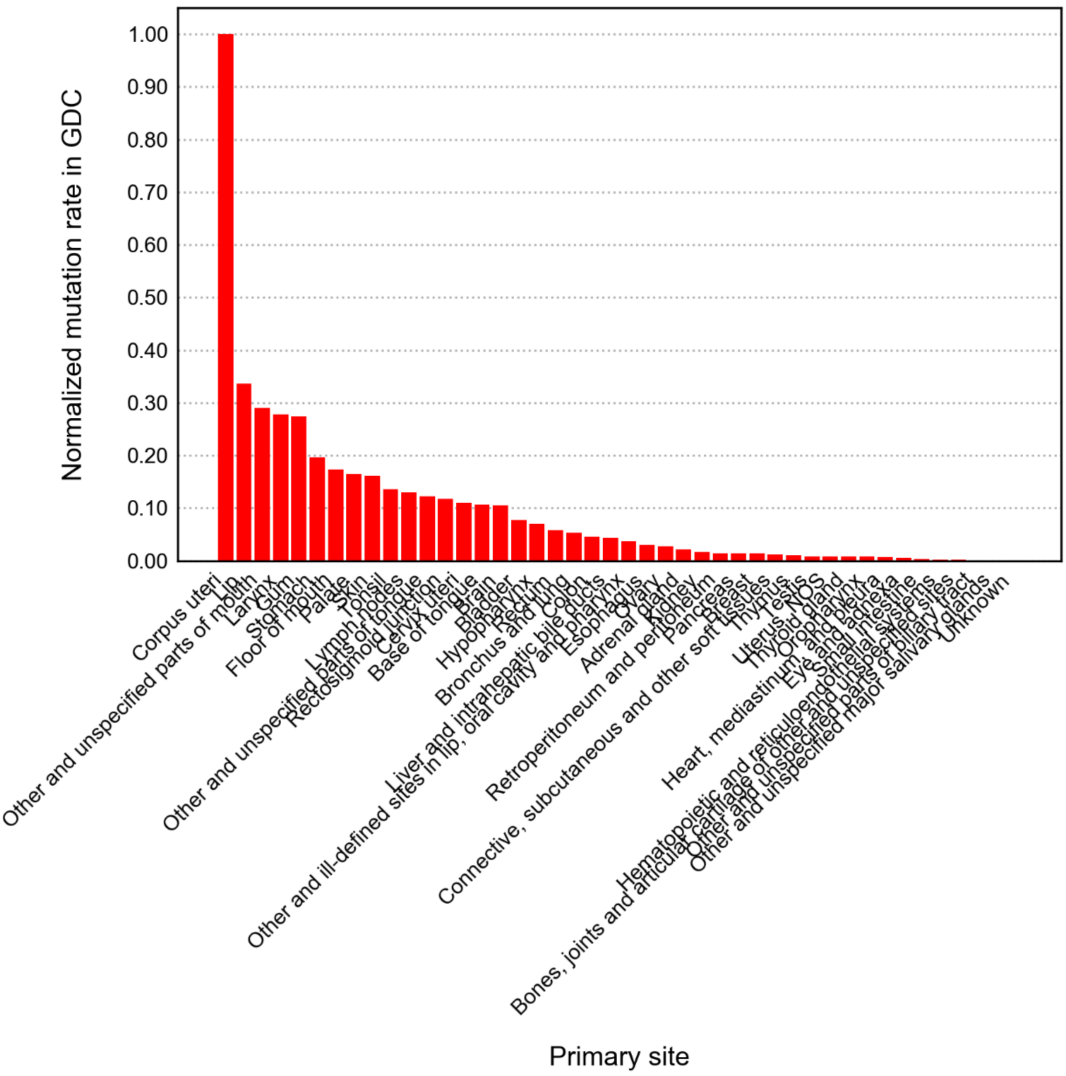

Supplementary figure 8. ***GPCR mutation rates by cancer type.*** *Normalized GPCR mutation rate per primary site (i.e. cancer type). Mutation rate per primary site is normalized by the number of patients in GDC with that cancer type.*

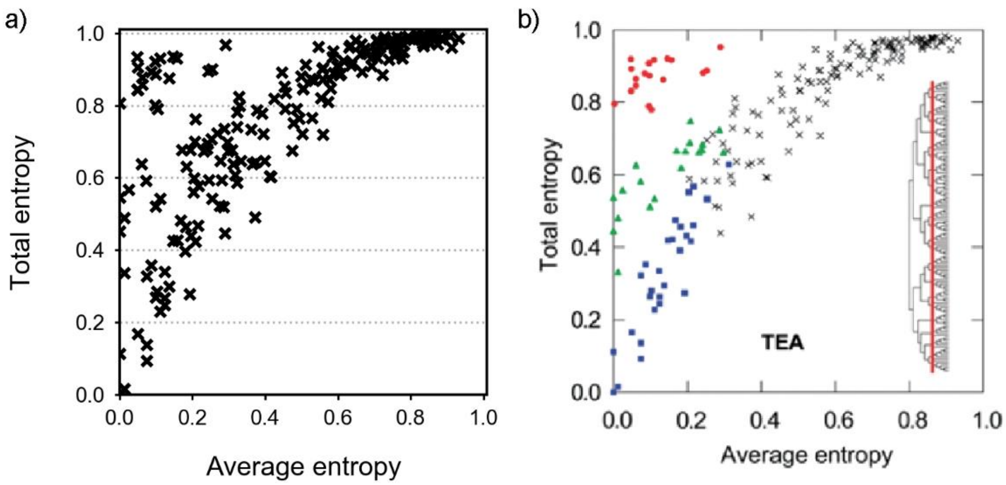

Supplementary figure 9. ***Two-entropy analysis re-implementation. (a)*** *Re-implementation of two-entropy analysis in a synthetic dataset as defined by Ye et al. in* [1]*.* ***(b)*** *Original analysis, figure adapted from Ye et al. in* [20]*.*
